# Methodological Issues Related to the Reported Association of A Body Shape Index With Abdominal Aortic Calcification in Patients With Hypertension

**DOI:** 10.1002/clc.70390

**Published:** 2026-06-22

**Authors:** Laiba Shahab, Syed Huzaifa Khan, Javeria Kashaf Zain

**Affiliations:** ^1^ Department of Medicine Nowshera Medical College Nowshera Pakistan; ^2^ Department of Medicine Khyber Medical University Peshawar Pakistan

To the Editor,

1

We have read with much interest the paper by Qiu and Wang, which appeared in Clinical Cardiology [1]. The researchers suggested that ABSI could predict the risk of AAC development and serve for cardiovascular risk stratification in hypertensive patients. We concur with both the novelty of the studied population as well as the significance of identifying AAC biomarkers. Nevertheless, we feel compelled to bring to your attention some issues regarding multicollinearity during the computation of ABSI, misinterpretation of the results of dose‐response modeling, and excessive importance attributed to ABSI as a predictor. Addressing these issues will help to better understand the significance of the obtained results.

First of all, it seems that there is some inconsistency related to the application of a specific regression model. Indeed, ABSI depends on BMI, waist circumference, and height of participants. Therefore, taking all of them as covariates when performing multiple regression analysis implies control for BMI and waist circumference. ABSI was designed to be mathematically independent of BMI and waist circumference and hence substitutable for them in regression analysis models, as shown by Krakauer and Krakauer [2]. In addition, Christakoudi et al. demonstrated that adjustment for BMI and waist circumference renders ABSI mathematically meaningless [3]. Furthermore, Midi et al. stated that the simultaneous inclusion of mathematically correlated covariates in regression models may bias and render unstable the estimates of their coefficients [4]. Consequently, conclusions about the independent predictive ability of ABSI cannot be drawn on the basis of such models, and the results should be reproduced using a properly constructed model.

Aside from the problem highlighted above, three further concerns render the results less reliable. To start with, the authors conclude that *p* > 0.05 in their restricted cubic spline analysis confirms a linear relationship between ABSI and AAC. However, a non‐significant result for nonlinearity simply indicates insufficient statistical power to detect a curved relationship, not confirmation of true linearity [5]. This is further contradicted by Yin et al. who, using the exact same NHANES 2013–2014 data set, reported a *U*‐shaped relationship between ABSI and AAC [6], directly challenging the linear claim of the target article. Second, the authors describe an AUC of 0.625 as having moderate discriminatory ability; however, by established standards in clinical prediction research, this value falls within the poor discrimination range [7]. Furthermore, no formal statistical comparison of AUC values was conducted using the DeLong test, the recommended method for comparing correlated AUC values [8]. Third, the TRIPOD guidelines, the international standard for prediction model reporting, require internal validation and benchmarking against existing models, neither of which was performed in this study [9]. Taken together, these concerns regarding collinearity, misinterpretation of dose‐response modeling, and overstatement of discriminative performance collectively raise important questions about the validity of the reported independence claim and the proposed clinical utility of ABSI for AAC risk stratification in hypertensive patients.

We respectfully invite the authors to address these methodological concerns, as doing so would substantially strengthen the scientific contribution of this work to the growing literature on anthropometric predictors of vascular calcification in hypertensive populations.

## Author Contributions

All authors have read and approved the final manuscript.

## Funding

The authors have nothing to report.

## Ethics Statement

The authors have nothing to report.

## Consent

The authors have nothing to report.

## Conflicts of Interest

The authors declare no conflicts of interest.

## Data Availability

The authors have nothing to report.
